# Comprehensive Model of Annual Plankton Succession Based on the Whole-Plankton Time Series Approach

**DOI:** 10.1371/journal.pone.0119219

**Published:** 2015-03-17

**Authors:** Jean-Baptiste Romagnan, Louis Legendre, Lionel Guidi, Jean-Louis Jamet, Dominique Jamet, Laure Mousseau, Maria-Luiza Pedrotti, Marc Picheral, Gabriel Gorsky, Christian Sardet, Lars Stemmann

**Affiliations:** 1 Sorbonne Universités, UPMC Univ Paris 06, UMR 7093 LOV, F-75005, Paris, France; 2 CNRS, UMR 7093 LOV, F-75005, Paris, France; 3 Sorbonne Universités, UPMC Univ Paris 06, UMR 7009 BioDev, F-75005, Paris, France; 4 CNRS, UMR 7009 BioDev, F-75005, Paris, France; 5 Université du Sud Toulon-Var, PROTEE EBMA, 83000, La Garde, France; Technical University of Denmark, DENMARK

## Abstract

Ecological succession provides a widely accepted description of seasonal changes in phytoplankton and mesozooplankton assemblages in the natural environment, but concurrent changes in smaller (*i*.*e*. microbes) and larger (*i*.*e*. macroplankton) organisms are not included in the model because plankton ranging from bacteria to jellies are seldom sampled and analyzed simultaneously. Here we studied, for the first time in the aquatic literature, the succession of marine plankton in the whole-plankton assemblage that spanned 5 orders of magnitude in size from microbes to macroplankton predators (not including fish or fish larvae, for which no consistent data were available). Samples were collected in the northwestern Mediterranean Sea (Bay of Villefranche) weekly during 10 months. Simultaneously collected samples were analyzed by flow cytometry, inverse microscopy, FlowCam, and ZooScan. The whole-plankton assemblage underwent sharp reorganizations that corresponded to bottom-up events of vertical mixing in the water-column, and its development was top-down controlled by large gelatinous filter feeders and predators. Based on the results provided by our novel whole-plankton assemblage approach, we propose a new comprehensive conceptual model of the annual plankton succession (*i*.*e*. whole plankton model) characterized by both stepwise stacking of four broad trophic communities from early spring through summer, which is a new concept, and progressive replacement of ecological plankton categories within the different trophic communities, as recognised traditionally.

## Introduction

The ecological succession is defined as an orderly and directional process of community development that is self-controlled, discontinuous, and shaped by the physical variability of the environment, leading to a stable ecosystem [1–3]. In dynamic systems such as coastal areas, lakes, and estuaries, it has been shown that plankton assemblages are likely to evolve over short time periods (*i*.*e*. seasons or shorter). They experience successive replacements of species caused by biotic interactions and abiotic forcing, until the system is reset to a pioneer state by exogenous forcing [4–8].

Successions of phyto- and mesozooplankton in temperate systems have been extensively studied over the past 30 years [9], but generally independently of each other. More studies have focused on phytoplankton compared to zooplankton, because changes in the latter are more difficult to document [10]. For phytoplankton communities, succession is generally initialized by intense vertical mixing, which causes nutrient replenishment of the photic layer. The community then evolves by replacements that are largely controlled by differential depletion of the various nutrients (for lakes, see [8], PEG model steps 9 to 15). The development of zooplankton populations follows the spring phytoplankton bloom, with successive appearance of grazers and predators. Finally, the combined effects of autumn water-column destabilization and reduction in day length brings the annual succession to its end.

Physical processes are known to play major roles in succession [7–9]. For phytoplankton, mixing stimulates growth and determines spatio-temporal patterns by changing nutrient and light availability. In contrast for zooplankton, the importance of physical processes is still unclear, but may largely depend on localized advection and meteorological events [10–11] and on indirect effects of mixing such as dilution of populations [12].

The traditional view of the replacement-based phytoplankton succession has been explicitly challenged, in a few publications, by authors who proposed the idea that diatoms were added to the initial small-phytoplankton community instead of replacing it [8, 13]. The possible extension of this idea to mesozooplankton has not been explored as yet, but it could be a key concept to study different components of the plankton and even whole-plankton assemblages. The latter include, in addition to phyto-, microzoo- and mesozooplankton, heterotrophic bacteria and macroplankton, both crustaceans (*e*.*g*. krill) and gelatinous organisms that can be filter feeding (essentially tunicates) or carnivorous (ctenophores and cnidarians). Bacteria or macroplankton have seldom been included in studies of whole-plankton assemblages [9], although they possibly mediate critical community reorganizations [14–16]. Bacteria, heterotrophic protists and macrozooplankton where included in the PEG succession model [9], although this integration was tentative because the authors relied on studies that did not cover the whole plankton.

The objective of the present study was to revisit the accepted model of plankton succession and examine the above emerging idea by confronting these hypotheses to a data series that would, for the first time in the literature, describe completely the changes occurring in a whole-plankton assemblage, from heterotrophic bacteria to macroplankton, not including fish and fish larvae for which no consistent data set was available. In order to obtain a realistic description of changes that took place in the natural environment, we conducted our observations over a multi-season period, *i*.*e*. 10 months, at relatively high frequency, *i*.*e*. weekly.

## Materials and Methods

### Ethic statement

Specific permission was not required to conduct sampling for this research.

The authors confirm that the field studies did not involve any endangered or protected species.

### Study Site

**Fig 1:**
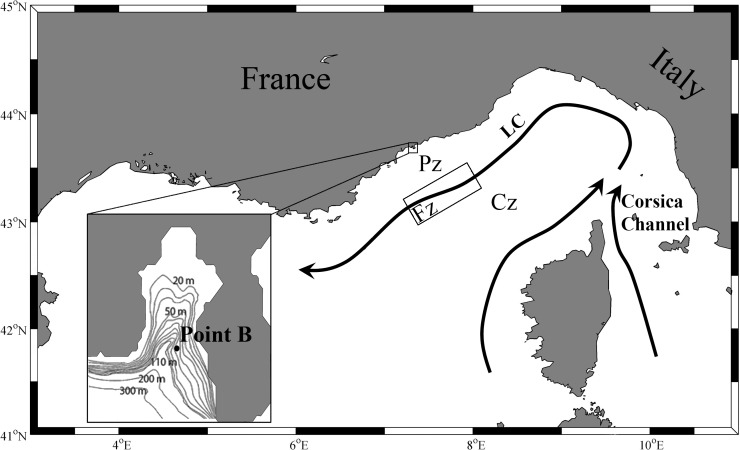
Map of the study area showing the general circulation in the Ligurian basin (black arrows). The peripheral zone (Pz), the frontal zone (Fz), schematized as a box superimposed on the top black arrow, and the central zone (Cz) are represented. LC stands for Ligurian Current. Bathymetric map of the Bay of Villefranche with the sampling station Point B (43°41.10 N, 7°19.00 E).

The sampling station (point B) is located at the entrance of the Bay of Villefranche-sur-Mer (43°41.10 N, 7°19.00 E; water depth ~85 m), on the northern coast of the Ligurian Sea, NW Mediterranean Sea (Fig. 1). It is characterized by low chlorophyll-*a* concentrations [17], and is part of the “intermittent blooming” eco-region of the Mediterranean Sea [18]. Water-column stratification varies from well mixed in winter to strongly thermally-stratified in summer, with transition periods in April-May and November-December. Deep waters from the Var Canyon (1 km offshore) can be upwelled into the bay [19], thus causing nutrient replenishment.

### Sampling

The sampling station was occupied weekly during ten months (*i*.*e*. 45 times) from December 2010 to October 2011. On each sampling date, one Conductivity Temperature Depth (CTD) profile was recorded using a SBE25 CTD. Water samples were collected at 6 different depths using Niskin bottles (0, 10, 20, 30, 50, and 75 m) for later analysis of nutrients (NO_3_ and SiO_4_) by colorimetry using a Technicon Alliance autoanalyzer, and for determination of picoplankton and nanoplankton (see below). For analyses of nanoplankton, subsamples from the 6 water samples were pooled into a single sample following the rule of trapezoidal integration, *i*.*e*. the volumes mixed were proportional to the sum of half the vertical distances from the sampling depth, above and below. Microplankton samples were collected with a 20 *μ*m mesh size plankton net (0.09 m² mouth opening) manually deployed from the side of the ship (vertical 75–0 m haul, 12 m.min^-1^). Zooplankton were sampled daily using two 75–0 m vertical net tows with a “WP2” 200 *μ*m mesh size net (0.25 m² mouth opening) for mesozooplankton, and a “Regent” 680 *μ*m mesh size net (0.785 m² mouth opening) for macroplankton. The daily samples from each net were pooled for each calendar week.

### Plankton analysis and classification into Plankton Ecological Categories (PECs)

**Fig 2:**
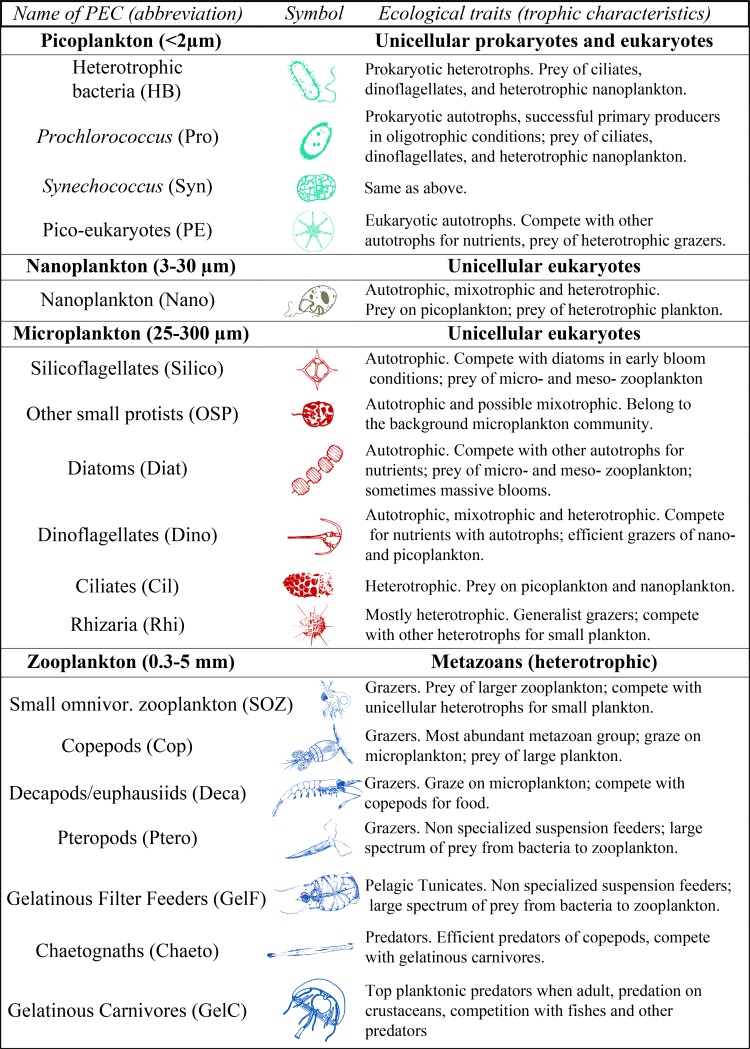
18 Plankton Ecological Categories, PECs (abbreviations), symbols, and ecological traits. The symbols used as visual aid in the figures.

In the literature, aggregated Plankton Functional Types (PFTs) have been defined within the context of biogeochemical modelling [20–21]. In the present study, we define Plankton Ecological Categories (PECs) by reference to ecological characteristics, in order to investigate ecological dynamics and interactions instead of biogeochemical fluxes. We successively applied three criteria: (1) size, *i*.*e*. picoplankton, nanoplankton, microplankton, and meso-macroplankton, (2) taxonomy, taking into account our ability to sample, analyze and identify the PEC quantitatively, and (3) ecological traits such as trophic behaviour, and relations with other PECs, *e*.*g*. prey-predator relationship or competition for a given resource. Hence, the 18 PECs summarized in Fig. 2 are more than a trophic classification as they consider sizes, life cycles, defense mechanisms, and times of occurrence at our sampling station while remaining taxonomically coherent.

The abundances of heterotrophic (*i*.*e*. bacteria) and autotrophic (*i*.*e*. cyanobacteria, and pico-eukaryotes) picoplankton (<2μm) were determined by flow cytometry [22]. Nanoplankton samples (here 3 to 30 *μ*m Equivalent Spherical Diameter, ESD) were preserved in Lugol’s iodine solution and imaged using an Olympus IX71 inverted microscope associated with a HD camera. Microplankton samples (here 25 to 300 *μ*m ESD) were preserved with Lugol’s iodine solution, and imaged with a FlowCam imaging instrument [23]. Meso- and macroplankton samples (300 to 5000 *μ*m ESD) were preserved in formalin, and digitized using a ZooScan imaging system [24]. Images generated by the three systems (*i*.*e*. inverted microscope, FlowCam, and ZooScan) were processed and analyzed using the ZooProcess software [24].

Four picoplankton PECs were determined from the flow cytograms. Vignettes of individual objects in the nano- to macro- plankton size fractions produced with the imaging instruments (*i*.*e*. inverted microscope, FlowCam, and ZooScan) were automatically classified prior to visual validation of identifications [24]. Approximately 500,000 plankton vignettes were extracted from nearly 1,000,000 output vignettes, the remaining vignettes being detrital, out of focus or artefact objects. The plankton vignettes were classified in 61 taxa using the Plankton Identifier software [25], and further grouped into 14 PECs. These plus the four picoplankton PECs made up 18 PECs (Fig. 2).

Each object had an associated size. A fixed size was used for each group of picoplankton: heterotrophic bacteria, 0.5 μm; *Prochlorococcus*, 0.6 μm; *Synechococcus*, 0.8 μm; pico-eukaryotes, 2 μm. Plankton individuals recognized by the three imaging systems were automatically sized during the analyses of images, and the size of each individual was converted to ESD to calculate the corresponding individual biovolume. Finally, the biovolume of each PEC (mm^3^) was estimated by adding up the biovolumes of all individual organisms in that PEC, and the result was referenced to the volume of seawater sampled (mm^3^.m^-3^).

### Data analyses

Water column stability was estimated using the Brunt-Vaïsälä frequency to identify the depth of the maximum density gradient (*i*.*e*. the pycnocline) [26].

Hierarchical Chronological Clustering [27] was used to identify coherent periods in the time series. “Physical” clusters were based on temperature (T) and salinity (S) profiles, thus representing the temporal sequence of physical changes in the water column. Prior to analysis, each T and S profile was averaged over 2-m depth intervals between 2 and 80 m. The resulting T and S profiles for each sampling day were combined to create a vector of 40 T data points followed by 40 S data points (total: 80 data points). The final data matrix had 45 rows (sampling weeks) x 80 columns (combined T and S data), and each of the 80 columns was a descriptor in the clustering analysis. “Biological” clusters were based on time series of log-transformed PEC biovolumes, thus representing the temporal sequence of changes in the plankton assemblage. The data matrix had 45 rows (sampling weeks) x 18 columns (PECs), and each of the 18 columns was a descriptor in the clustering analysis.

Indicator Values (*IV*) were used to identify indicator PECs for each biological cluster [28]. Indicator Values (*IV*, eq. 3) are computed by multiplying a measure of specificity (*S*, eq. 1) by a measure of fidelity (*F*, eq. 2). *S* corresponds to the ratio between the average biovolume of PEC *i* in cluster *j*, and the sum of the mean biovolume of the PEC *i* in all clusters (eq. 1). *F* is the ratio between the number of sampling dates where PEC *i* in cluster *j* was present and the total number of sampling dates in this cluster (eq. 2).

$$S=\frac{N_{PEC_{ij}}}{N_{PEC_{i}}}$$ (1)

$$F=\frac{N_{DATES_{ij}}}{N_{DATES_{j}}}$$ (2)

$$IV_{ij}=S\times F\times 100$$ (3)

*IV*s were calculated for each PEC according to the biological clustering levels identified determined by the Hierarchical Chronological Clustering, and sorted from the most to the less indicative. At each clustering level, each PEC was allocated to a single cluster. Finally, the significance of *IV*s (p-value, i.e. probability that the allocation of each PEC to a cluster was random) was tested by bootstrap, using 10,000 random permutations of dates between clusters.

## Results

### Seasonal variations of the water column

**Fig 3:**
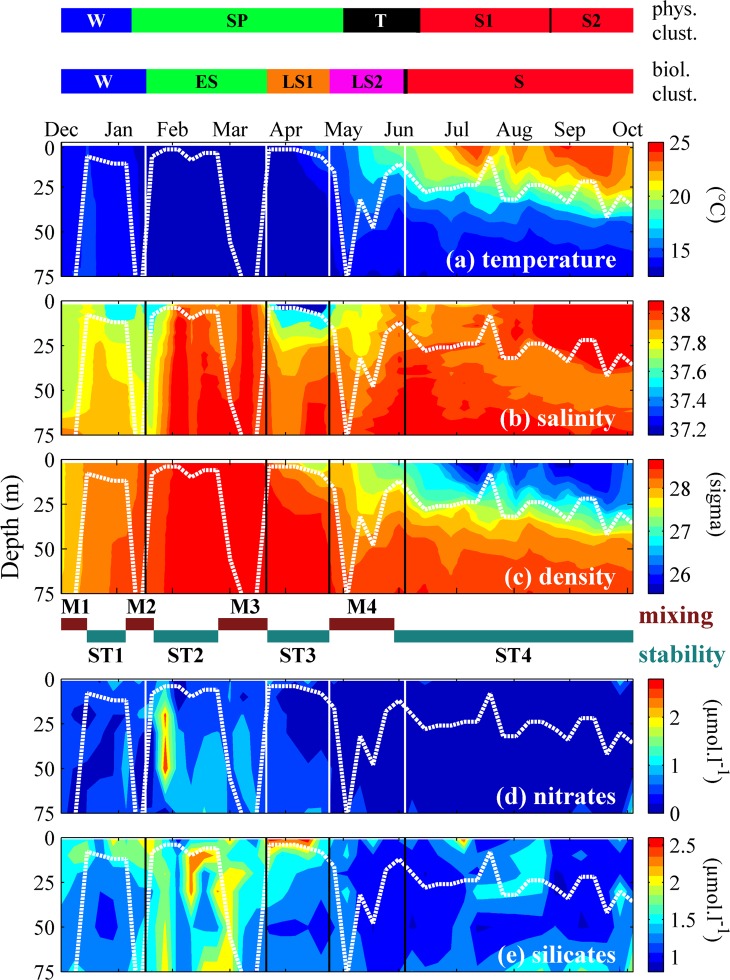
Variations of environmental conditions at the sampling station between December 2010 and October 2011. The two coloured bars at the top represent the duration of the different clusters based on the two sets of variables, *i*.*e*. physical (W = winter, SP = spring, T = transition, S1 = summer 1, S2 = summer 2) and biological (W = winter, ES = early spring, LS1 = late spring 1, LS2 = Late Spring 2, S = summer) (the clustering analyses are detailed in Fig. 4). Panels: (a) temperature, (b) salinity, (c) water density with (indicated below) periods of mixing (M1–M4) and stability (ST1-ST4), (d) nitrate, and (e) silicate. The white dotted curve repeated in all panels represents the depth of the maximum density gradient (pycnocline) estimated from the Brunt-Vaïsälä frequency. The vertical lines represent the limits of clusters based on biological variables (*i*.*e*. PEC time series).

**Fig 4:**
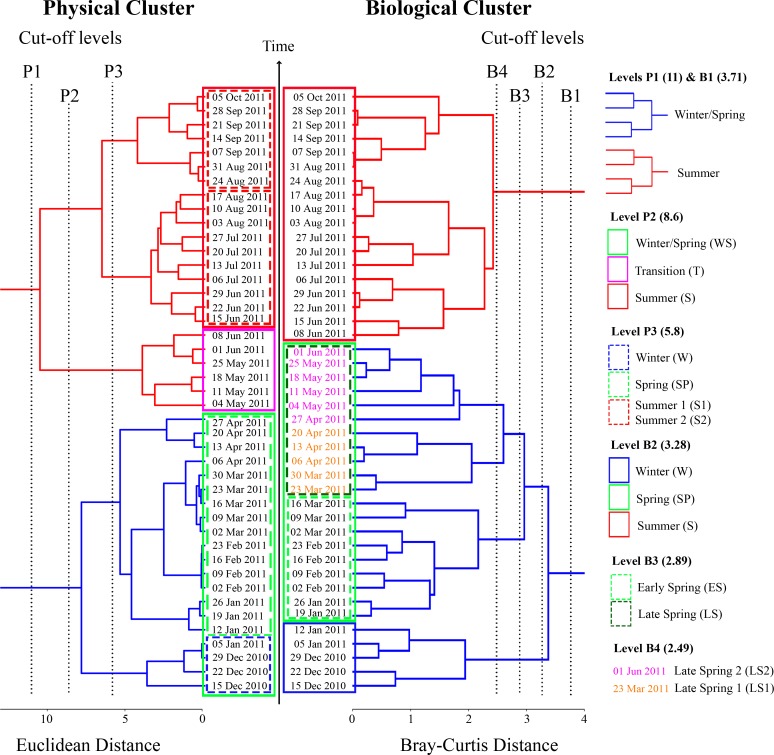
Cluster analyses of physical (temperature and salinity, left) and biological (PEC biovolumes, right) data. Three significant clustering levels were identified for the physical characteristics of the water column: level P1, (cut-off = 11, red and blue branches of the tree); level P2 (cut-off = 8.6, solid-line coloured rectangles); level P3 (cut-off = 5.8, dashed-line coloured rectangles). Four significant clustering levels were identified for the biological data: level B1 (cut-off = 3.71, red and blue branches of the tree); level B2 (cut-off = 3.28, solid-line coloured rectangles); level B3 (cut-off = 2.89, dashed-line rectangles) which clustered 4 groups; level B4 (cut-off = 2.89, coloured fonts) which clustered 5 groups.

The dynamics of the water column are represented in Fig. 3. Three levels of partitioning were identified through the clustering analysis based on temperature and salinity. Winter/spring profiles were separated from summer profiles at the first level of partitioning (Fig. 4, clustering level P1). Water-column temperature decreased from winter to early spring, and remained homogenous until the beginning of April, after which it increased at surface to reach ~15°C in early May; this characterized the onset of thermal stratification, which persisted throughout the summer months (Fig. 3a). There were two episodes of lower salinity at surface, the first occurring in January, and the second at the end of March when low salinity propagated from surface (~37.2) to bottom (~37.9) (Fig. 3b). During the winter/spring period, the depth of the pycnocline varied between the surface and below 75 m, and showed four marked peaks corresponding to four mixing events (M1 to M4), *i*.*e*. time intervals during which vertical mixing occurred, separated by periods of stability (ST1 to ST4) (Fig. 3c). The duration and timing of the various periods of mixing and stability are given in Table 1. The concentrations of nitrate and silicate were high in winter and spring, started to decrease in late April, and were minima throughout summer (Fig. 3d, 3e). Nutrient concentrations increased threefold after M2, simultaneously with a sudden decrease of water-column temperature and salinity.

**Table 1:** Dates of mixing events and stability periods.

	Start	End
M1	01-Dec-2010	15-Dec-2010
ST1	15-Dec-2010	05-Jan-2011
M2	05-Jan-2011	20-Jan-2011
ST2	20-Jan-2011	24-Feb-2011
M3	24-Feb-2011	22-Mar-2011
ST3	22-Mar-2011	25-Apr-2011
M4	25-Apr-2011	06-Jun-2011
ST4	06-Jun-2011	05-Oct-2011

At the second clustering level (Fig. 4, clustering level P2), a transition cluster appeared in May and early June, corresponding to the onset of thermal stratification. It marked out the transition between the cold and often vertically mixed water column in winter/spring and the warm and stratified conditions in summer.

At the third clustering level (Fig. 4, clustering level P3), there were five clusters: winter, spring, transition, summer 1 and summer 2. Winter conditions were separated from the colder, nutrient-rich water column in spring by mixing event M2. Spring was separated from the transition cluster by mixing event M3, and the transition cluster corresponded to mixing event M4. The summer cluster was partitioned in two periods, *i*.*e*. summer 1 and summer 2, and the latter was characterized by the highest annual surface salinities and temperatures. The dates of the different clusters are given in Fig. 4.

### Seasonal variations of the whole-plankton assemblage

**Fig 5:**
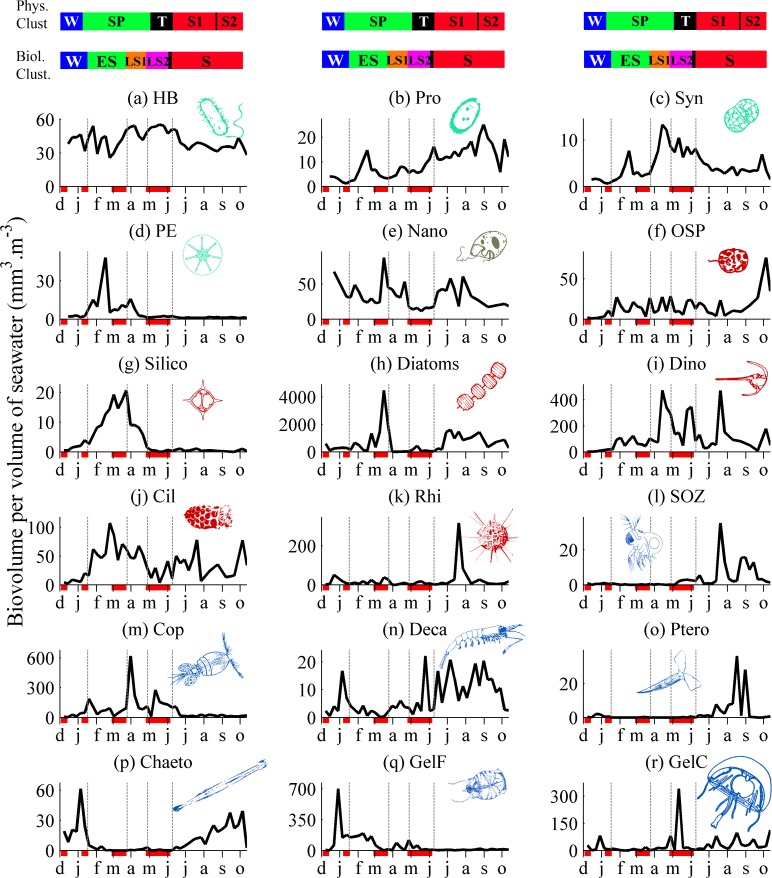
Time series of PEC biovolumes at the sampling station from December 2010 to October 2011. The top coloured bars represent the periods identified from the clustering analyses based on physical and biological variables (coloured bars as in Fig. 3). The vertical dashed lines represent limits of clusters based on biological variables (*i*.*e*. PEC time series). Red dashes under the *x*-axes represent mixing events (see Fig. 3). Note the different scales on the *y*-axes.

Two long periods were identified in the biological (*i*.*e*. PECs) time series at the first clustering level (Fig. 4, clustering level B1). They corresponded chronologically to the winter/spring and summer seasons, with distinct PEC assemblages (Fig. 5). In this study, we refer to four trophic communities, *i*.*e*. the microbial community, primary producers, grazers, and predators (Table 2). The winter/spring community consisted of an initial assemblage of primary producers (*i*.*e*. pico-eukaryotes, silicoflagellates, *Synechococcus* and dinoflagellates, as shown by the *IV* analysis, S1 Table, to which diatoms could be associated as they showed their highest peak in spring, (Fig. 5h), and an initial assemblage of grazing and filter-feeding animals (*i*.*e*. copepods, gelatinous filter feeders and ciliates, S1 Table). The summer community was essentially composed of a second assemblage of primary producers (*i*.*e*. diatoms and *Prochlorococcus*, S1 Table), a second assemblage of primary consumers (*i*.*e*. small omnivorous zooplankton, decapods/euphausiids and pteropods, S1 Table), and an assemblage of predators (*i*.*e*. chaetognaths and gelatinous carnivores, S1 Table) that had been much less present in winter/spring (Fig. 5).

**Table 2:** Distribution of the 18 PECs among the 4 trophic communities used in the text.

*Trophic communities*	*PECs*
Microbial community	Heterotrophic bacteria (HB), *Prochlorococcus* (Pro), *Synechococcus* (Syn), Nanoplankton (Nano) Other small protists (OSP), Ciliates (Cil), Rhizaria (Rhi)
Primary producers	*Prochlorococcus* (Pro), *Synechococcus* (Syn), Pico-eukaryotes (PE), Silicoflagellates (Silico), Diatoms (Diat), Dinoflagellates (Dino)
Grazers	Dinoflagellates (Dino), Ciliates (Cil), Small omnivorous zooplankton (SOZ), Copepods (Cop), Decapods/euphausiids (Deca), Pteropods (Ptero), Gelatinous Filter Feeders (GelF),
Predators	Chaetognaths (Chaeto), Gelatinous carnivores (Gel)

The second clustering level (Fig. 4, clustering B2) partitioned the long winter/spring period into winter and spring periods. The winter community was mostly characterized by heterotrophic organisms, *i*.*e*. chaetognaths and gelatinous filter feeders, nanoplankton, which can be autotrophic and heterotrophic, and Rhizaria, which can be strictly heterotrophic or host autotrophic symbionts (S1 Table). Chaetognaths and gelatinous filter feeders showed simultaneous high peaks just before the end of the winter (based on a single sampling date), and the biovolume of gelatinous filter feeders (mostly large tunicates) remained relatively high (although progressively declining) between M2 and M3 (Fig. 5p, 5q). At that time, small primary producers (*i*.*e*., pico-eukaryotes and silicoflagellates) started to grow one after the other (Fig. 5d, 5g). The spring community was characterized by an assemblage of primary producers (silicoflagellates, pico-eukaryotes, *Synechococcus* and dinoflagellates) associated with an assemblage of primary consumers (ciliates and copepods) (S1 Table). The summer community identified at clustering level B2 was similar to that at clustering level B1, except that chaetognaths and Rhizaria were indicators of summer at clustering level B1 and of winter at clustering level B2. However these two PECs both showed high biovolumes in summer, and could thus be also associated with the summer cluster.

The third clustering level (Fig. 4, clustering level B3) partitioned the spring cluster into periods of early and late spring. The winter and summer PEC communities remained unchanged relative to clustering levels 1 and 2 (S1 Table). The early spring community was characterized by silicoflagellates, pico-eukaryotes and ciliates. As diatoms showed their highest peak during this period, they could also be associated with the early spring cluster. The late spring community was characterized by *Synechococcus*, dinoflagellates, copepods and heterotrophic bacteria. The fourth clustering level (Fig. 4, clustering level B4) partitioned the B3-level late spring cluster at the date of the physical transition from winter/spring to summer (see above). The first resulting B4-level cluster was characterized by three of the four PECs identified for the B3-level late spring cluster (*i*.*e*. *Synechococcus*, dinoflagellates, and copepods), and the second cluster by the fourth PEC (*i*.*e*. heterotrophic bacteria) (S1 Table).

Components of the microbial community (Table 1) were present throughout the sampling period (Fig. 5a, 5c, 5e, 5f, 5j, 5k) in each cluster at all clustering levels. At clustering level B1 winter/spring was characterized by heterotrophic bacteria, *Synechococcus*, nanoplankton and ciliates, and summer by *Prochlorococcus*, Rhizaria and other small protists, (S1 Table). At clustering levels B2, B3 and B4, winter was characterized by Rhizaria and nanoplankton, and summer by *Prochlorococcus* and other small protists (S1 Table). At clustering level B2, spring was characterized by ciliates, *Synechococcus* and heterotrophic bacteria. At clustering levels B3 and B4, early spring was characterized by ciliates, and late spring by *Synechococcus* and heterotrophic bacteria (S1 Table).

In summary: (1) Eukaryotic primary producers appeared in early spring and followed the replacement sequence pico-eukaryotes—silicoflagellates—diatoms—dinoflagellates—diatoms. (2) Gelatinous filter feeders were present in winter, decreased strongly in early spring, and were replaced by copepods in spring, which were themselves replaced by small omnivorous zooplankton and decapods/euphausiids during the summer. (3) Predators (chaetognaths) were present in winter, disappeared in spring, and reappeared in summer. (4) Components of the microbial community were present in each cluster at all clustering levels.

## Discussion

### Local hydrology and its influence on plankton dynamics

In the Ligurian basin, the general circulation is alongshore (Ligurian Current, LC, Fig. 1), with little cross-shore transport [29]. A permanent yet variable geostrophic front [30, 31] isolates the homogenous coastal waters (Peripheral zone, Pz) from the Frontal zone (Fz) and the offshore Central zone (Cz). Earlier studies have shown that the Fz was responsible for discontinuities in the main physical and biological characteristics between the Pz and the Cz [30, 32–34], and that ecological processes at our sampling site were representative of the Pz waters [30, 33, 34]. During most of our sampling period, the Fz was located between 5 and 20 km offshore [35], and it is thus assumed that the plankton community that we sampled mostly or totally belonged to the coastal Pz. Alongshore transport of varying plankton communities could also affect the sampling station, but we could not test this hypothesis directly because there are no biological data available elsewhere in the coastal Ligurian Sea. However, climatic analysis showed that the whole Ligurian Sea is under similar large scale forcing [36]. A comparison of salinity time series at the sampling station [37] and at the offshore DYFAMED site [38] showed similar seasonal patterns with low salinity in spring (April-June) and autumn (October-December) and high salinity in summer (July-September) and winter (January-March) as a result of the seasonal precipitation regime [38]. This indicates that the seasonal climatic forcing is similar over the whole Ligurian basin, including the Pz. Under such conditions, it can be hypothesized that the along-shore spatial biological gradients were weak. Hence it is expected that the along-shore transport of plankton, which we expect to occur in the Ligurian basin, did not influence much the annual plankton succession at our sampling site.

### Initiation of succession: bottom-up and top-down controls

The initiation of the plankton succession is generally thought to result from the input of nutrients driven by vertical mixing, followed by some stabilisation of the water column [4, 8, 9, 39]. This sequence of events is likely what we observed at our sampling station after the period of strong vertical mixing that occurred during the second half of January (Fig. 3c, M2), when the vertically stabilised water column showed high nutrient concentrations and rapid, but limited increase in the biovolumes of pico-eukaryotes and silicoflagellates.

From M2 to M3, the biovolume of gelatinous filter feeders (mostly large tunicates) was relatively high, (Fig. 5q), perhaps explaining the low increase in phytoplankton biovolumes during that period, despite the stabilisation of the water column and the availability of nutrients, by top-down grazing control of phytoplankton by gelatinous filter feeders. Indeed, the grazing pressure exerted by these organisms can represent 50% to 100% of daily primary production in some coastal areas [40, 41]. A brief, strong bloom of diatoms followed the collapse of gelatinous filter feeders during M3 (Fig. 5h, 5q), which is also consistent with the idea that these organisms top-down controlled the biomass of phytoplankton. This corresponds to the occurrence of short and intense diatom blooms in the Bay of Villefranche [42–44], especially when the abundances of gelatinous filter feeders were very low in late winter or early spring [45]. Similarly, overwintering populations of gelatinous filter feeders have been reported to prevent phytoplankton accumulation in offshore areas of the Southern Ocean [46, 47], the north-eastern US coast [48], and the Mexican Baja Californian coast [41]. It is likely that the mixing of the water column during M3 induced a local input of saltier, nutrient rich waters in the water column from the bottom (Fig. 3d, 3b). The combination of nutrient input and increased daylight (March) have probably favoured the diatom bloom, even though the water column was not stratified yet.

It follows that the initiation of the plankton succession was not only controlled from the bottom up by the establishment of favourable abiotic conditions, *i*.*e*. nutrient replenishment by vertical mixing and increased daylight [5, 8, 9], but also by the release of top-down control, *i*.*e*. grazing by gelatinous filter feeders. The two effects were related to episodes of strong mixing followed by stabilisation of the water column.

### Succession process: stepwise transitions controlled by vertical mixing and macroplankton

The annual plankton succession started during the second half of January, after M2. The nutrient replenishment of the water column that followed M2 was concomitant with successive increases in pico-eukaryote and silicoflagellate biovolumes in February (Fig. 5d, 5g), and was followed by a 10-fold increase in the biovolume of diatoms in March (Fig. 5h). This sequence was typical of the spring phytoplankton bloom [8].

The spring bloom was followed by peaks in copepod and dinoflagellate biovolumes. It is hypothesized that the first major copepod peak, just after M3 (Fig. 5m), could have benefited from the development of diatoms. It could have also been favoured by the progressive decline of gelatinous filter feeders between mixing events M2 to M3, which would have released part of their grazing pressure on phytoplankton, thus reducing the competition between copepods and gelatinous filter feeders for the phytoplankton resource, and also decreased the predation of gelatinous filter feeders on copepod eggs [49, 50]. One hypothesis for the stepwise collapse of gelatinous filter feeders is that it was caused by water-column mixing. Indeed, the two steps of GelF decline were simultaneous with mixing events. According to Behrenfeld [12] mixing could have a detrimental effect on consumers by diluting the population in the deeply mixed water column. It is hypothesized that these combined factors could have enabled the transition from the phytoplankton-dominated community to the more complex herbivorous grazing community (S1 Table, Clustering level B3). The observed changes from the heterotrophic winter community were as follows: a stepwise decrease of gelatinous filter feeders from mixing events M2 to M3, the phytoplankton bloom in early spring, and the establishment of copepods and dinoflagellates in late spring. The latter resembles the mature, final stage of the plankton succession described by Margalef [4].

The biological transition period (Fig. 4, clustering level B4, LS2) ended with the fourth mixing event, M4. This period saw the rapid decline of copepods (Fig. 5m), possibly as a combined consequence of the decline of diatoms, competition for food with dinoflagellates [51], and potentially accrued predation pressure by cnidarians [52]. After M4, macroplankton carnivores (chaetognaths and cnidarians) and smaller diatoms developed simultaneously, and copepods were replaced by small omnivorous zooplankton and decapods/euphausiids. The unexpected summer increase of diatoms coupled with low biovolumes of crustacean grazers and high biovolumes of gelatinous predators suggests a top-down control of crustacean grazers by carnivorous gelatinous plankton and chaetognaths, which would have allowed the development of diatoms, which resembles a typical trophic cascade pattern [53]. In addition, an ongoing study at our sampling station shows that the abundance of fish larvae, which are notoriously planktivorous [9], increased during the summer (Robin Failletaz, pers. comm.), which strengthens the present top-down control hypothesis. It is therefore hypothesized that the onset of summer stable physical conditions enabled the emergence of an extra phase of the plankton succession. As documented for long-term fisheries trends [54] and lakes [8, 9], the trophic cascade could have contributed to maintain high functional diversity in the whole-plankton assemblage, *i*.*e*. the co-existence of microbial, phytoplankton, grazer, and predator PECs.

### Ecological succession: progressive stacking up of trophic communities

**Fig 6:**
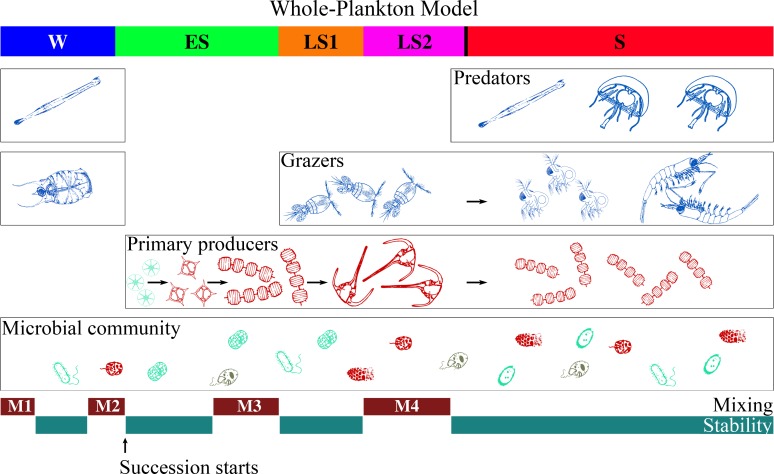
Conceptual representation of the whole-plankton model. The top coloured bar represents the periods identified from the clustering analysis based on biological variables (*i*.*e*. PEC time series; coloured bar as in Fig. 3). Each trophic community is represented in a horizontal box, and the boxes are stacking up from bottom to top. In the primary producers and grazers boxes, the black arrows represent the traditional replacement-based ecological succession. The stacking of trophic boxes represents the stacking of trophic level occurring over the course of the succession. Each group of organisms depicted here is defined in Fig. 2. The bottom bars identify the periods of vertical mixing and stability of the water column.

Components of the microbial community were present in each cluster at all clustering levels (Fig. 5 and S1 Table), showing the persistence of this community at the sampling station over the year, as already documented by Ferrier-Pages & Rassoulzadegan [55]. Heterotrophic bacteria, *Prochlorococcus*, *Synechococcus*, nanoplankton and other small protists showed relatively low variability over time when compared with other PECs (Fig. 5). This indicates that the microbial community is a persistent background assemblage, on which the communities of larger plankton organisms develop when environmental conditions become favourable. This idea extends to the whole plankton assemblage the proposal of Landry [56] and Barber & Hiscock [13] that phytoplankton succession could develop over a microbial background (Fig. 6).

The succession of primary producers started with the M2 mixing event in mid-January. After M2, small eukaryotic phytoplankton developed, were replaced after M3 by blooming large diatoms, which were themselves replaced by dinoflagellates, which were finally replaced by small diatoms. This corresponds to the phytoplankton succession described by Margalef [4, 57]. In offshore waters, the onset of favourable nutrient, light and stability conditions leads to an increase in the photosynthetic ability and specific growth rate of diatoms [58]. As the bloom matures, diatoms progressively make up most of the phytoplankton biomass [56, 59, 60]. The large increase in the proportional abundance of diatoms relative to the non-diatom phytoplankton has long been interpreted as a replacement of the pre-bloom community by diatoms [4]. Although this interpretation is widely accepted, some studies have noted that not only diatoms but also smaller primary producers such as *Prochlorococcus* and *Synechococcus* benefit from the favourable conditions and grow during the bloom [13, 56]. In other words, the small primary producers, which are part of the microbial community, would be not replaced by diatoms during the blooms, but the latter would instead be added to the existing phytoplankton community, whose biomass they rapidly dominate [13, 56, 61]. In our study, autotrophic organisms did not replace the microbial community described in the previous paragraph, but they were instead “stacked up” over it (Fig. 6). The usually recognized replacement of PECs over the course of the succession took place within the primary production community.

After the M3 mixing event, crustacean grazers showed up. The first to appear were copepods, which were later replaced by small omnivorous zooplankton (essentially cladocerans) and decapods/euphausiids. This pattern is consistent with observations in some major coastal areas [11, 39] and fresh waters [8]. The grazing community was stacked up over primary producers, in the same way as the latter had been stacked up over the microbial community (Fig. 6). There was replacement of PECs within the grazing community.

Finally, populations of some predators (*i*.*e*. Chaetognaths) started to develop in mid-summer and persisted until winter, whereas others (*i*.*e*. gelatinous carnivores, mostly small medusa and siphonophores) occurred at various times during the year but were mainly present in summer. The predators were stacked up over the grazing community, in the same way as the latter had been stacked up over the primary production community (Fig. 6).

## Conclusions

The remarkable match between physical and biological events in our time series adds to the existing body of evidence that hydrodynamics play key roles in shaping plankton succession. In addition to these bottom up effects, our unique whole-plankton series, which covers organisms 5 orders of magnitude in size from heterotrophic bacteria to gelatinous macroplankton, identified the top-down control exerted by macroplankton gelatinous filter feeders at the start of the succession process, at the transition from the spring bloom to the post bloom state. In addition, macroplankton predators may have promoted an extra phase in the succession *via* a trophic cascade.

Based on the original results provided by our novel whole-plankton assemblage approach, we proposed the first comprehensive model of the annual plankton succession (*i*.*e*. whole-plankton model, Fig. 6), which was characterized by both: (1) stepwise stacking of four broad trophic communities from microbes to predators, from early spring through summer, which is a new concept, and (2) progressive replacement of ecological plankton categories (*i*.*e*. PECs) within the different trophic communities, as recognised traditionally. This shows that the traditional view of the annual plankton succession, as plankton categories replacing each other over the year, is correct within different trophic communities (*e*.*g*. primary producers or grazers). However, because trophic communities had been generally studied separately and not as whole-plankton assemblages, the traditional view missed the key point that the different trophic communities are stacked up over each other during the course of the succession, thus building up an increasingly complex food web.

## Supporting Information

S1 Table Indicator values (*IV*) outputs for each of the 4 biological clustering levels, for 1 every PEC. Clusters are identified with the same terminology and colour coding as in Figs. 1, 2 and 3. *IVs* and significance levels of p-values are shown besides each plankton group. 3 Groups, *IV* and p-values printed in italics are non-significant. *, ** and *** stand for *p*<0.05, *p*<0.01, and *p*<0.001, respectively.
(PDF)

## References

[1] Odum E. Strategy of ecosystem development. Science. 1969; 164: 262–270. 10.1126/science.164.3877.262 5776636

[2] Odum E. Emergence of ecology as a new integrative discipline. Science. 1977; 195: 1289–1293. 10.1126/science.195.4284.1289 17738398

[3] Allen TFH, Bartell SM, Koonce JF. Multiple stable configurations in ordination of phytoplankton community change rates. Ecology. 1977; 58: 1076–1084. 10.2307/1936927

[4] Margalef R. Temporal succession and spatial heterogeneity in natural phytoplankton In: Buzzati-Traverso A, editor. Perspectives in Marine Biology. Los Angeles : University of California Press; 1958 pp. 323–349.

[5] Margalef R. 1967 Some concepts relative to the organization of plankton. In: Allen & Unwin, editors Oceanography and Marine Biology: An Annual Review. Aberdeen: Aberdeen University Press; 1967 pp. 257–289.

[6] Margalef R. Perspectives in ecological theory. 1st ed Chicago: University of Chicago Press; 1968.

[7] Levasseur M, Therriault J, Legendre L. Hierarchical control of phytoplankton succession by physical factors. Mar Ecol Prog Ser. 1984; 19: 211–222. 10.3354/meps019211

[8] Sommer U, Gliwicz Z, Lampert W, Duncan A. The Peg-Model of seasonal succession of planktonic events in fresh waters. Arch Hydrobiol. 1986; 106: 433–471.

[9] Sommer U, Adrian R, Domis L, Elser J, Gaedke U, Ibelings B, et al Beyond the Plankton Ecology Group (PEG) model: mechanisms driving plankton succession In: Futuyma DJ, editor. Annual Review of Ecology, Evolution, and Systematics 43. Palo Alto: Annual Reviews; 2012 pp. 429–448.

[10] Ribera D’Alcala MR, Conversano F, Corato F, Licandro P, Mangoni O, Marino D, et al Seasonal patterns in plankton communities in a pluriannual time series at a coastal Mediterranean site (Gulf of Naples): an attempt to discern recurrences and trends. Sci Mar. 2004; 68: 65–83. 10.3989/scimar.2004.68s165

[11] Calbet A, Garrido S, Saiz E, Alcaraz M, Duarte CM. Annual zooplankton succession in coastal NW Mediterranean waters: the importance of the smaller size fractions. J Plankton Res. 2001; 23: 319–331. 10.1093/plankt/23.3.319

[12] Behrenfeld MJ. Abandoning Sverdrup’s Critical Depth Hypothesis on phytoplankton blooms. Ecology. 2010; 91: 977–989. 10.1890/09-1207.1 20462113

[13] Barber RT, Hiscock MR. A rising tide lifts all phytoplankton: Growth response of other phytoplankton taxa in diatom-dominated blooms. Glob Biogeochem Cycles. 2006; 20: GB4S03 10.1029/2006GB002726

[14] Verity PG, Smetacek V. Organism life cycles, predation, and the structure of marine pelagic ecosystems. Mar Ecol Prog Ser. 1996; 130: 277–293. 10.3354/meps130277

[15] Hay S. Marine ecology: Gelatinous bells may ring change in marine ecosystems. Curr Biol. 2006; 16: 679–682. 10.1016/j.cub.2006.08.010 16950089

[16] Richardson AJ, Bakun A, Hays GC, Gibbons MJ. The jellyfish joyride: causes, consequences and management responses to a more gelatinous future. Trends Ecol Evol. 2009; 24: 312–322. 10.1016/j.tree.2009.01.010 19324452

[17] Bustillos-Guzman J, Claustre H, Marty JC. Specific phytoplankton signatures and their relationship to hydrographic conditions in the coastal Northwestern Mediterranean sea. Mar Ecol Prog Ser. 1995; 124: 247–258. 10.3354/meps124247

[18] D’Ortenzio F, Ribera D’ Alcala MR. On the trophic regimes of the Mediterranean Sea: a satellite analysis. Biogeosciences. 2009; 6: 139–148

[19] Nival P, Corre M. Annual variation of surface hydrology in bay of Villefranche-Sur-Mer. Ann Inst Oceanogr. 1976; 52: 57–78.

[20] Le Quere C, Harrison SP, Prentice IC, Buitenhuis ET, Aumont O, Bopp L, et al Ecosystem dynamics based on plankton functional types for global ocean biogeochemistry models. Glob Change Biol. 2005; 11: 2016–2040. 10.1111/j.1365-2468.2005.01004.x

[21] Hood RR, Laws EA, Armstrong RA, Bates NR, Brown CW, Carlson C, et al Pelagic functional group modeling: Progress, challenges and prospects. Deep-Sea Res Part II-Top Stud Oceanogr. 2006; 53: 459–512. 10.1016/j.dsr2.2006.01.025

[22] Marie D, Brussaard C, Partensky F, Vaulot D. Flow cytometric analysis of phytoplankton, bacteria and viruses In John Wiley & Sons editors. Current Protocols in Cytometry. 1999 11.11.1–11.11.15.

[23] Sieracki CK, Sieracki ME, Yentsch CS. An imaging-in-flow system for automated analysis of marine microplankton. Mar Ecol Prog Ser. 1998; 168: 285–296. 10.3354/meps168285

[24] Gorsky G, Ohman MD, Picheral M, Gasparini S, Stemmann L, Romagnan JB, et al Digital zooplankton image analysis using the ZooScan integrated system. J Plankton Res. 2010; 32: 285–303. 10.1093/plankt/fbp124

[25] Gasparini S, Antajan E. 2013 Oct 15 [cited 03 February 2015]. Plankton Identifier. Available: http://www.obs-vlfr.fr/~gaspari/Plankton_Identifier/index.php

[26] Mann KH, Lazier JRN. Dynamics of marine ecosystems: biological-physical interactions in the oceans. 3rd ed Malden, MA: Blackwell Publishing; 2006.

[27] Legendre P, Dallot S, Legendre L. Succession of species within a community—chronological clustering, with applications to marine and fresh-water zooplankton. Am Nat. 1985; 125: 257–288. 10.1086/284340

[28] Dufrene M, Legendre P. Species assemblages and indicator species: The need for a flexible asymmetrical approach. Ecol Monogr. 1997; 67: 345–366. 10.2307/2963459

[29] Millot C. Circulation in the Western Mediterranean Sea. J Mar Syst. 1999; 20: 423–442. 10.1016/10.1016/S0924-7963(98)00078-5

[30] Sournia A, Brylinski J, Dallot S, Lecorre P, Leveau M, Prieur L, et al Hydrological fronts off the coasts of france—a review. Oceanol Acta. 1990; 13: 413–438.

[31] Piterbarg L, Taillandier V, Griffa A. Investigating frontal variability from repeated glider transects in the Ligurian Current (North West Mediterranean Sea). J Mar Syst. 2014; 129: 381–395. 10.1016/j.jmarsys.2013.08.003

[32] Boucher J, Ibanez F, Prieur L. Daily and seasonal variations in the spatial distribution of zooplankton populations in relation to the physical structure in the Ligurian Sea front. J Mar Res. 1987; 45: 133–173.

[33] Molinero JC, Ibanez F, Souissi S, Bosc E, Nival P. Surface patterns of zooplankton spatial variability detected by high frequency sampling in the NW Mediterranean. Role of density fronts. J Mar Syst. 2008; 69: 271–282. 10.1016/j.jmarsys.2005.11.023

[34] Stemmann L, Prieur L, Legendre L, Taupier-Letage I, Picheral M, Guidi L, et al Effects of frontal processes on marine aggregate dynamics and fluxes: An interannual study in a permanent geostrophic front (NW Mediterranean). J Mar Syst. 2008; 70: 1–20. 10.1016/j.jmarsys.2007.02.014

[35] Ferraris M, Berline L, Lombard F, Guidi L, Elineau A, Mendoza-Vera JM, et al Distribution of *Pelagia noctiluca* (Cnidaria, Scyphozoa) in the Ligurian Sea (NW Mediterranean Sea). J Plankton Res. 2012; 34: 874–885. 10.1093/plankt/fbs049

[36] Goberville E, Beaugrand G, Sautour B, Treguer P. Climate-driven changes in coastal marine systems of western Europe. Mar Ecol Prog Ser. 2010; 408: 129–148. 10.3354/meps08564

[37] Garcia-Comas C, Stemmann L, Ibanez F, Berline L, Mazzocchi MG, Gasparini S, et al Zooplankton long-term changes in the NW Mediterranean Sea: Decadal periodicity forced by winter hydrographic conditions related to large-scale atmospheric changes? J Mar Syst. 2011; 87: 216–26. 10.1016/j.jmarsys.2011.04.003

[38] Marty JC, Chiaverini J. Hydrological changes in the Ligurian Sea (NW Mediterranean, DYFAMED site) during 1995–2007 and biogeochemical consequences. Biogeosciences. 2010; 7: 2117–28. 10.5194/bg-7-2117-2010

[39] Jamet JL, Jean N, Boge G, Richard S, Jamet D. Plankton succession and assemblage structure in two neighbouring littoral ecosystems in the north-west Mediterranean Sea. Mar Freshw Res. 2005; 56: 69–83. 10.1071/MF04102

[40] Andersen V. Salp and pyrosomid blooms and their importance in biogeochemical cycles In: Bone Q, editor. The biology of pelagic tunicates. Oxford: Oxford University Press; 1998 pp 125–137.

[41] Hereu CM, Lavaniegos BE, Gaxiola-Castro G, Ohman MD. Composition and potential grazing impact of salp assemblages off Baja California during the 1997–1999 El Nino and La Nina. Mar Ecol Prog Ser. 2006; 318: 123–140. 10.3354/meps318123

[42] Claustre H, Marty JC, Cassiani L. Intraspecific differences in the biochemical-composition of a diatom during a spring bloom in Villefranche-Sur-Mer bay, Mediterranean-Sea. J Exp Mar Biol Ecol. 1989; 129: 17–32. 10.1016/0022-0981(89)90060-9)

[43] Fernex FE, Braconnot JC, Dallot S, Boisson M. Is ammonification rate in marine sediment related to plankton composition and abundance? A time-series study in Villefranche Bay (NW Mediterranean). Estuar Coast Shelf Sci. 1996; 43: 359–371. 10.1006/ecss.1996.0075

[44] Gómez F, Gorsky G. Annual microplankton cycles in Villefranche Bay, Ligurian Sea, NW Mediterranean. J Plankton Res. 2003; 25: 323–339. 10.1093/plankt/25.4.323

[45] Licandro P, Ibanez F, Etienne M. Long-term fluctuations (1974–1999) of the salps *Thalia democratica* and *Salpa fusiformis* in the northwestern Mediterranean Sea: Relationships with hydroclimatic variability. Limnol Oceanogr. 2006; 51: 1832–1848.

[46] Dubischar CD, Bathmann UV. Grazing impact of copepods and salps on phytoplankton in the Atlantic sector of the Southern ocean. Deep-Sea Res Part II-Top Stud Oceanogr. 1997; 44: 415–433. 10.1016/S0967-0645(96)00064-1

[47] Bernard KS, Steinberg DK, Schofield OME. Summertime grazing impact of the dominant macrozooplankton off the Western Antarctic Peninsula. Deep-Sea Res Part I-Oceanogr Res Pap. 2012; 62: 111–122. 10.1016/j.dsr.2011.12.015

[48] Madin LP, Kremer P, Wiebe PH, Purcell JE, Horgan EH, Nemazie DA. Periodic swarms of the salp *Salpa aspera* in the Slope Water off the NE United States: Biovolume, vertical migration, grazing, and vertical flux. Deep-Sea Res Part I-Oceanogr Res Pap. 2006; 53: 804–819. 10.1016/j.dsr.2005.12.018

[49] Haskell AGE, Hofmann EE, Paffenhofer GA, Verity PG. Modeling the effects of doliolids on the plankton community structure of the southeastern US continental shelf. J Plankton Res. 1999; 21: 1725–1752. 10.1093/plankt/21.9.1725

[50] Deibel D, Lowen B. A review of the life cycles and life-history adaptations of pelagic tunicates to environmental conditions. ICES J Mar Sci. 2012; 69: 358–369. 10.1093/icesjms/fsr159

[51] Sherr EB, Sherr BF. Heterotrophic dinoflagellates: a significant component of microzooplankton biomass and major grazers of diatoms in the sea. Mar Ecol Prog Ser. 2007; 352: 187–197. 10.3354/meps07161

[52] Sabates A, Pages F, Atienza D, Fuentes V, Purcell JE, Gili JM. Planktonic cnidarian distribution and feeding of *Pelagia noctiluca* in the NW Mediterranean Sea. Hydrobiol. 2010; 645: 153–165. 10.1007/s10750-010-0221-z

[53] Sommer U. Trophic cascades in marine and freshwater plankton. Int Rev Hydrobiol. 2008; 93: 506–516. 10.1002/iroh.200711039

[54] Casini M, Hjelm J, Molinero JC, Lovgren J, Cardinale M, Bartolino V. Trophic cascades promote threshold-like shifts in pelagic marine ecosystems. Proc Natl Acad Sci USA. 2009; 106: 197–202. 10.1073/pnas.0806649105 19109431 PMC2629246

[55] Ferrier-pages C, Rassoulzadegan F. Seasonal impact of the microzooplankton on picoplankton and nanoplankton growth-rates in the Northwest Mediterranean sea. Mar Ecol Prog Ser. 1994; 108: 283–294. 10.3354/meps108283

[56] Landry MR. Integrating classical and microbial food web concepts: evolving views from the open-ocean tropical Pacific. Hydrobiol. 2002; 480: 29–39. 10.1023/A:1021272731737

[57] Margalef R. Life-forms of phytoplankton as survival alternatives in an unstable environment. Oceanol Acta. 1978; 1: 493–509.

[58] Falkowski PG, Laws EA, Barber RT, Murray JW. Phytoplankton and their role in primary, new, and export production In: Fasham MJR, editor. Ocean Biogeochemistry: The Role of the Ocean Carbon Cycle in Global Change. New York: Springer; 2003 pp. 99–120.

[59] Landry MR, Constantinou J, Latasa M, Brown SL, Bidigare RR, Ondrusek ME. Biological response to iron fertilization in the eastern equatorial Pacific (IronEx II). III. Dynamics of phytoplankton growth and microzooplankton grazing. Mar Ecol Prog Ser. 2000; 201: 57–72. 10.3354/meps201057

[60] Sarthou G, Timmermans KR, Blain S, Tréguer P. Growth physiology and fate of diatoms in the ocean: a review. J Sea Res. 2005; 53: 25–42. 10.1016/j.seares.2004.01.007

[61] Ryther JH. Components of Ecosystems In: Riley G, editor. Marine Biology I. Washington D.C; 1963 pp. 25.

