# Supplementary material for: Comprehensive Model of Annual Plankton Succession Based on the Whole-Plankton Time Series Approach
Source: PLoS One. 2015 Mar 17;10(3):e0119219. doi: 10.1371/journal.pone.0119219 (PMC4363592; doi:10.1371/journal.pone.0119219)
Supplement: S1 Table — Clusters are identified with the same terminology and colour coding as in Figs. 1, 2 and 3. IVs and significance levels of p-values are shown besides each plankton group. 3 Groups, IV and p-values printed in italics are non-significant. *, ** and *** stand for p<0.05, p<0.01, and p<0.001, respectively. (PDF) [file pone.0119219.s001.pdf]

Table S1 - Indicator values (*IV*) outputs for each of the 4 biological clustering levels, for every PEC. Clusters are identified with the same terminology and colour coding as in Figs. 1, 2 and 3. *IVs* and significance levels of p-values are shown besides each plankton group. Groups, *IV* and p-values printed in italics are non-significant. *, ** and *** stand for *p*<0.05, *p*<0.01, and *p*<0.001, respectively.

**Clust. Level B1**				**Clust. Level B2**				**Clust. Level B3**				**Clust. Level B4**			
B1=5.8		*IV*	*p*	B2=3.28		*IV*	*p*	B3=2.89		*IV*	*p*	B4=2.49		*IV*	*p*
**Winter/Spring**	Silico	80.9	***	**Winter**	Chaeto	50.0	***	**Winter**	Chaeto	46.5	***	**Winter**	Chaeto	43.1	***
	PE	69.1	***		GelF	48.1	**		GelF	36.3	**		GelF	30.3	*
	GelF	63.2	***		Rhi	37.8	**		Rhi	29.6	***		Rhi	25.8	***
	Cop	59.3	***		Nano	36.1	***		Nano	27.3	***		Nano	22.0	***
	Syn	51.4	***	**Spring**	Silico	52.6	*	**Early Spring**	Silico	49.9	***	**Early Spring**	Silico	38.6	***
	HB	51.3	***		PE	46.5	***		PE	42.1	**		PE	33.5	**
	Dino	50.2	**		Syn	44.8	*		Cil	31.3	**		Cil	24.7	**
	Cil	50.2	***		Dino	41.3	**	**Late Spring**	Syn	38.0	***	**Late Spring**	Syn	28.3	**
	Nano	*50.1*	*0.15*		Cop	39.8	**		Dino	31.5	*		*Cop*	*25.2*	*0.07*
**Summer**	Ptero	87.2	***		Cil	38.7	**		Cop	30.8	**		Dino	24.2	***
	SOZ	*79.7*	*0.91*		HB	34.1	***		HB	26.3	***	**LS2**	HB	21.0	*
	Chaeto	*72.6*	*0.91*	**Summer**	SOZ	64.7	**	**Summer**	Ptero	61.4	***	**Summer**	Ptero	60.0	***
	Deca	*63.1*	*0.12*		*Ptero*	*63.4*	*0.31*		*SOZ*	*56.9*	*0.24*		*SOZ*	*49.7*	*0.09*
	GelC	*60.5*	*0.96*		*Pro*	*45.9*	*0.05*		*Deca*	*35.4*	*0.06*		Deca	28.6	*
	Pro	59.5	**		Deca	43.2	*		Pro	34.3	***		Pro	27.4	**
	Diat	*56.4*	*0.60*		GelC	41.6	***		GelC	33.6	***		GelC	26.6	**
	Rhi	55.3	***		OSP	40.0	***		Diat	29.6	***		Diat	25.6	***
	OSP	53.2	***		Diat	38.7	**		OSP	28.8	***		OSP	22.6	***
